# GeneSeer: A sage for gene names and genomic resources

**DOI:** 10.1186/1471-2164-6-134

**Published:** 2005-09-21

**Authors:** Andrew J Olson, Tim Tully, Ravi Sachidanandam

**Affiliations:** 1 Cold Spring Harbor Laboratory, Cold Spring Harbor, NY, USA

## Abstract

**Background:**

Independent identification of genes in different organisms and assays has led to a multitude of names for each gene. This balkanization makes it difficult to use gene names to locate genomic resources, homologs in other species and relevant publications.

**Methods:**

We solve the naming problem by collecting data from a variety of sources and building a name-translation database. We have also built a table of homologs across several model organisms: *H. sapiens*, *M. musculus*, *R. norvegicus*, *D. melanogaster*, *C. elegans*, *S. cerevisiae*, *S. pombe *and *A. thaliana*. This allows GeneSeer to draw phylogenetic trees and identify the closest homologs. This, in turn, allows the use of names from one species to identify homologous genes in another species. A website  is connected to the database to allow user-friendly access to our tools and external genomic resources using familiar gene names.

**Conclusion:**

GeneSeer allows access to gene information through common names and can map sequences to names. GeneSeer also allows identification of homologs and paralogs for a given gene. A variety of genomic data such as sequences, SNPs, splice variants, expression patterns and others can be accessed through the GeneSeer interface. It is freely available over the web  and can be incorporated in other tools through an http-based software interface described on the website. It is currently used as the search engine in the RNAi codex resource, which is a portal for short hairpin RNA (shRNA) gene-silencing constructs.

## Background

*"Biologists would rather share their toothbrush than share a gene name"*:Michael Ashburner [1]. Biologists use a variety of names for genes, based on their specialization. It is a daunting task to use gene names to locate resources such as sequences or publications. For example:

1. *ABCA4*, *ABC10*, *ABCR*, *FFM*, *RMP*, *RP19*, *STGD*, *STGD1 *are all names for the same gene; ATP binding cassette, sub family A (ABC1).

2. The same gene name can be written in different ways; *cyclinD1 *versus *cyclin D1*.

3. Researchers modify names; *hPRL *is used to denote the human form of *PRL*.

4. Names can be species specific. For example, human *p53 *is called *tumor protein 53 (Li-Fraumeni syndrome)*, whereas in mouse it is called *transformation related protein 53 (trp53)*.

5. Names can be specialization specific. The same gene is known as *PUF60 *in the pre-mRNA splicing field and *FIR *in the transcription field. It is also known by the names *RoBPI *and *siah-bp*. The *D. melanogaster *field knows it as *hfp*. To add to the confusion, yeast has a family of proteins called *PUF *that have similar function but are unrelated in terms of sequence homology.

In order to locate genomic resources for a given gene using a familiar name, a reference name has to first be identified from GenBank (or other databases such as Swiss-Prot, TrEMBL [2] or ENSEMBL [3]). This reference name can then be used to access resources from a variety of databases (GenBank, ENSEMBL etc.). For example, once [GenBank:NM_000546] is identified as one of the reference names for p53, then sequences and other resources can be easily accessed in GenBank. But a search for p53 in the nucleotide database at NCBI [4] results in a list of more than 5700 accessions (over 285 pages), which necessitates manual curation to find the specific accessions of interest. It is possible to narrow down the search using advanced search features, but is error-prone and inconvenient, requiring several trials before a search can be fine-tuned.

There are attempts being made to streamline and standardize the naming process through the HUGO gene nomenclature committee (HGNC [5]). Unfortunately, there are historic names from different fields and scientists still tend to use fanciful names (especially in the *D. melanogaster *field, where names such as *crossbronx*, *disco-related *etc. are quite common).

Thus, there is a need for a tool that allows accessing information through names that are familiar to biologists from different sub-fields. In addition, analysis of large datasets creates the need for a programming interface that allows a program to access resources and accessions. Once a programming interface is designed, a website can be designed quite easily, to provide user-friendly access to the programming interface through cgi scripts and present results in an aesthetic and ergonomic fashion.

There are other tools and approaches that partially solve some of these problems and these are discussed and compared to GeneSeer in the discussion section.

## Implementation

GeneSeer retrieves and stores synonyms for the model organisms: *H. sapiens*, *M. musculus*, *R. norvegicus*, *D. melanogaster*, *C. elegans*, *S. cerevisiae*, *S. pombe *and *A. thaliana *from the following sources, GenBank [4], FlyBase [6], ExPASy [2,7], HUGO [5], ENSEMBL [3], UCSC [8], and Gene Ontology [9]. It also holds sequences for proteins and nucleotides as well as related information (splice variants, expression patterns etc.) about the genes. Similarities (homologies) between proteins are pre-calculated and stored in GeneSeer.

This paper is organized with the description of the software tools used in GeneSeer coming first followed by the algorithm and the underlying architecture.

### Software tools

There are three critical software tools we use, a database server, a genomic sequence server and a viewer to display alignments.

### Database

We store most of the data (except for sequence data) in a MySQL relational database [10]. There are separate tables for synonyms from each data source and an auxiliary table for synonyms from data sources such as micro-arrays or RNAi libraries. The data management is described in more detail in the name-translation section below.

### Genome packer (Gpacker)

GeneSeer needs to be able to frequently access sections of sequences from the sequence database. A tool called Gpacker was developed to allow fast random access to any of the SOFAR (described below) sequences or assembled genomic sequences. Gpacker uses a binary system to store sequence information, using 4 bits for each base in nucleotide data (a DNA sequence requires only 2 bits per position, but if SNP data is included, then 4 bits are necessary) and 8 bits for each amino acid in protein data (to account for the 20 possible amino acids). The binary files are indexed, with the indices stored in a database. This allows for fast random access of sequences.

### Light weight genome viewer (lwgv)

Many genes exhibit alternative splicing, with several splice variants created from a single locus. In addition, there are annotations of features such as repeats, SNPs, CpG islands etc. The light weight genome viewer (**lwgv**) is used to display the annotations of features at a given genomic locus. This tool also allows navigation to other resources on the web, such as NCBI [4] and UCSC [8], *lwgv *was developed for in-house use and is now publicly available at *Source Forge *[11].

### Architecture of GeneSeer

GeneSeer has three key features, the SOFAR database, name translation tables, and homology tables. SOFAR is a non-redundant collection of transcript sequences in each genome, whose construction is described below. The name-translation tables connect synonyms to each other. The homology tables contain pre-computed similarity scores for all pairs of proteins between and within species from a non-redundant collection, based on SOFAR.

GeneSeer is designed as a hub-and-spokes system, with the SOFAR database serving as the hub and the connections to resources and names serving as spokes. Every name or sequence that is submitted gets translated to a SOFAR name, either directly via the synonym tables, or indirectly through a BLAST [12] search of the SOFAR sequence database or through the use of the homology tables. SOFAR members are linked to resources and information, both internal and external to GeneSeer. We describe the three components of this architecture below.

#### SOFAR – Set Of FastA Representatives

*Entrez Gene *[13] (formerly *LocusLink) *provides an indexing for coding regions of the genome. However, this indexing is not complete, since there are regions that have cDNAs associated with them, which are not in Entrez Gene. Another feature that would be useful, but not provided by Entrez Gene, is a set of non-redundant accessions to represent each locus. The term **locus **is used here as a synonym for a coding region of the genome.

We built a set of mRNA accessions that includes the known genes and expert-curated cDNAs called SOFAR for each organism to overcome these problems. SOFAR is the key to GeneSeer's ability to return a concise set of results for any search.

The SOFAR database for an organism starts with one coding sequence. Each subsequent coding sequence that is considered for addition to the database gets checked for similarity to sequences already in SOFAR, through a BLAST [12] search, and gets added only if it is sufficiently different. We use a criterion of 60% similarity as a cutoff for entry into SOFAR.

The order in which sequences are considered for inclusion in SOFAR is crucial. In the case of human and mouse genomes, ordered gene lists are created by first using genes from RefSeq [14], NCBI's reference sequence resource, then sequences from Entrez Gene loci but not in RefSeq and then sequences that are expert annotated but are not in Entrez Gene, such as some kinases [15] and cDNAs for other functional groups. RefSeq annotates genes according to the reliability of the underlying evidence, for example, *validated *ranks higher than *predicted*. This is used to order the RefSeq genes amongst themselves. In case of all else being equal, the sequences are ordered by length (longest first).

**Figure 6:**
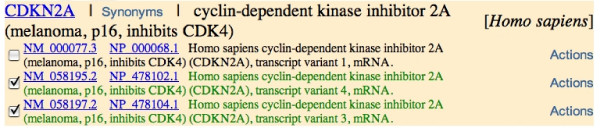
**Result of a search for INK4A in *H. sapiens***. In the results that are returned for each locus, all the RefSeq [14] accessions at the locus are returned, but the ones in SOFAR are highlighted in green. If a locus does not have a RefSeq accession, then only the accessions in SOFAR are returned. In this case the locus is CDKN2A [16] with three accessions, two of which, [GenBank:NM_058195] (ARF) and [GenBank:NM_058197] (INK4A), are from SOFAR and highlighted in green. This is discussed in the text.

The arbitrary 60% similarity cutoff can cause two kinds of mis-identifications,

1. Two similar sequences that are from different loci get assigned to the same locus, while they should have separate entries in SOFAR, e.g. duplicated genes such as *FUT5 *and *FUT6 *which occur on different loci on human chromosome 19.

2. Two sequences that are from the same locus can get identified as being sufficiently different from each other and get separate entries in SOFAR (e.g. *INK4a *and *ARF *are the same Entrez Gene, *CDKN2A *[16], figure 6).

Most of the cases from the first type can be resolved using Entrez Gene indices, however there remain eighty-nine cases of SOFAR members from multiple loci, all of which are immunoglobulin genes. For example, [GenBank:M14158] and [GenBank:D86998] are from different loci, but both are genomic sequences that contain parts of an immunoglobulin gene. On searching for them in GeneSeer, it tries to BLAST these sequences against SOFAR and comes up with unrelated genes. GeneSeer is not very useful for accessing resources for genes from this family. IMGT, the international ImMunoGeneTics information system [17,18] with its specialized sequence (IMGT/LIGM-DB) and gene (IMGT/GENE-DB [19]) databases is better suited for this purpose.

It is important to note that the two genes INK4a and ARF [16] are sufficiently different that they warrant inclusion as separate entries, since they have distinct sequences and functions. In *H. sapiens *there are 1130 loci which have multiple SOFAR members. This is not a problem, as the SOFAR members are different from each other and we want SOFAR to contain all possible non-redundant coding sequences.

As an additional step, unique sequences from splice variants are then entered to ensure that SOFAR contains every possible transcribed 25-mer.

A SOFAR database has been built for each of the organisms in GeneSeer. In the case of *D. melanogaster*, the set of FBgn numbers provided by *FlyBase *[6], which are a unique set of genes similar to *Entrez Gene*, was used to create the SOFAR database.

Almost every SOFAR member has a corresponding protein, except in the case of partial coding sequences, predicted genes and non-coding RNAs. These proteins can be used to construct a SOFAR set of proteins, which is used to generate data for the homology tables and viewer described below.

The SOFAR database is useful for other purposes, such as the design of RNAi hairpin constructs that can silence single genes [20]. Designs were checked against the SOFAR database to ensure that they did not match (with up to 2 mismatches) more than one sequence in the database.

#### Name translations

Each of the following databases, HUGO [5], ExPASy [2,7] (Swiss-Prot and TREMBL names), ENSEMBL [3], GenBank [4], FlyBase [6] and Gene Ontology [9], comes with a list of synonyms, which were downloaded and entered into a separate table for each dataset. The Genbank data contains a mapping from gene names to Entrez Gene IDs. The bulk of the names in GeneSeer are taken directly from a file provided at NCBI's ftp site [21]. FlyBase provides additional synonyms. The set of names is extended even further by extracting names from the definition lines of sequences which cross-reference an *Entrez Gene ID*.

Information relevant to the individual mRNA accessions, such as coding sequences (CDS) and protein domains, is extracted from GenBank flat files and stored in additional tables. The system utilizes the Entrez Taxonomy database [22,23] for translating between taxonomy ids and organism names. Up-to-date Gene Ontolgy (**GO**) [9] terms and associations are also incorporated, which allows users to search for genes by GO terms. To find GO terms for genes, it is easier to use tools such as GObar [24] or AMIGO [25]. Tables of genomic alignments provided by UCSC [8] are directly imported to the GeneSeer database. These alignments are currently used to help visualize the exon-intron structure of the genes.

An auxiliary translation table is used to store names that might be specific to particular datasets such as short hairpin names from the publicly available RNAi libraries or probe names from microarrays.

The database tables remain current through regular updates. The active GeneSeer database is replaced once every month, after a new version of the database is built and tested.

Each name gets mapped to a SOFAR representative. If an accession or EST name is submitted to the system and it is not recognized, then GeneSeer downloads the sequence and uses BLAST against SOFAR to identify its name. If this fails, then a mapping to the genome is used to identify the closest locus that contains a SOFAR representative. There are cases where everything fails and nothing is returned, such cases have to be curated manually, as they are usually ESTs that might not be reliable. Everytime such a translation succeeds, the result gets cached for fast response the next time around. The cached results will not survive an update to the GeneSeer system.

#### Homology tables and viewer

GeneSeer can identify homologs across species and present a phylogenetic tree. A matrix of similarity scores, based on BLAST [12], is pre-calculated for all pairs of SOFAR proteins in the system. The construction of the SOFAR protein database is described above. This matrix is used to generate clusters of related proteins. The clusters are aligned, using ClustalW [26], when the user requests a tree. A phylogenetic tree is then created from this multiple alignment using PHYLIP [27], which has been modified to run in batch mode, to build rooted and unrooted trees. A custom program renders the tree in *scalable vector graphics *(SVG) format to allow user interaction. This tree is not the same as one that would be derived from a careful alignment of domains and might be less accurate, but it definitely allows quick identification of close homologs. The results of the homology viewer can be a starting point for a more detailed phylogenetic analysis of the proteins in a family.

The homology viewer can be accessed using the *Action *menu item, *explore-homologs*, in the results page of a GeneSeer search. The result of clicking on this link is a page that allows fine-tuning the search parameters, or eliminating a species that might not be of interest. If the threshold of BLAST similarity scores is set lower, then fewer homologs will be considered while drawing the phylogenetic tree. Limiting the cluster sizes is important as the rendering of the phylogenetic tree can take a long time if there are too many members in the family, leading to a time-out error from the browser.

Sometimes it is not possible to reach a protein in a distant species directly. In such cases, it may be possible to use an intermediate organism to make the connection, that is, the intermediate organism's protein has homology to proteins in both species of interest. An *expand *checkbox in the fine-tuning page described above, allows such an exploration.

## Results

The GeneSeer server [28] can be accessed either using a web-browser or through a programming interface that is described below.

Gene names can be entered by hand or uploaded in the form of files containing lists. Sequences can also be uploaded in the form of fasta files. Results can be downloaded in the form of excel spreadsheets or text files and can be used to access information from NCBI, or to access splice variants and homologs. Tissue specificity of mRNA expression can also be accessed.

Since GeneSeer is accessible over http, programs can be written in almost any language to use it. We have plans to improve programmatic access, especially using semantic-web [29] based technologies such as Resource Description Framework(RDF), but improvements will be driven primarily by user-feedback and the needs of the community.

• To retrieve the SOFAR name for genes named p53 in csv format, use the URL, .

• To retrieve a list of homologs for the human gene named p53 (gene id 7157) use the URL, .

• To retrieve a list of genes with either the symbol p53 or p21 in the human genome (taxonomy id = 9606) in html format use the URL, .

### GeneSeer search features

GeneSeer is flexible in the kinds of names that can be used for searching. Thus, searches can be conducted using **Gene Symbols/Names, Keywords **(partial terms such as *casp *for caspase), **Keyword Symbols **(partial symbols, such as *erb *for erbb2), **OMIM ids **(online mendelian inheritance in man [30]), diseases/disorders (such as diabetes), **Tissue specificity **(tissue expression patterns, such as genes expressed in muscle, derived from UniGene [31]), **Gene Accessions, Protein Accessions, Entrez Gene IDs **(from Entrez Gene [13]), **UniGene Cluster IDs **(from UniGene [31]), **CDD Domain IDs **(from conserved domain database [32]), **Gene Ontology IDs **(from Gene Ontology [9]), **Definitions **(from definition lines in GenBank [4]), **HUGO IDs **(from IDs defined by HUGO [5]), **ENSEMBL IDs **(from IDs defined by ENSEMBL [3]), **SNPs **(from dbSNP [33]) and **Sequences **(nucleic acid and protein sequences).

Search terms can be entered either individually or in a comma-separated list or by uploading files (e.g. Excel spreadsheets, fasta files, or simple text files) containing the list of terms.

The easiest option is to first use the automatic search mode. The software will try to guess what the user has provided (accession, symbol etc.) and return its best answer. The automatic mode uses a restrictive search first, such as a name search, and iteratively expands the types of searches it performs, and stops searching when it finds a result. If this is unsatisfactory, then the **Go Further **button can improve results. The *Go Further *button will continue a few more methods and return more ambiguous results. If the results continue to be unsatisfactory, then one of the specialized modes (listed above) will need to be used and modifying the search terms might also help. For example, if *caspase2 *does not return a result, a search with *casp *as a keyword will return results that will definitely include *caspase 2*.

Results are displayed on a webpage but can also be downloaded to a comma-separated-value (csv) file using the *download .xls *operation provided on the results page. The csv file can be opened in a spreadsheet program or a text editor. If possible, the returned results always show the *Entrez Gene IDs *for each name. GeneSeer can be used to translate names into IDs for use in other programs which prefer to use Entrez Gene IDs, such as GObar [24].

In addition, on the results page, NCBI [4] pages for the search results can be accessed. Each individual result also has an associated *Action *link, that allows exploration of **PubMed **[34], for papers related to the item), **tissues, UCSC **(the genome browser at UCSC for the relevant genomic region) [8], **explore_homology **(view an approximate phylogeny of related genes), and **visualize gene **(visualize the genomic region and study splice variants).

If GeneSeer fails to find any of the terms, they are listed on the results page. The failures can be re-analysed using different search methods (searching by "keyword", "symbol" or other variations). As a last resort, a bug report can be sent via the website and a human curator will resolve the issue.

Complicated queries using the *search history *button can also be performed: an example is the search for all caspases that are expressed in the human brain, which is explained below. A list of searches done using any computer are stored on the server, and can be accessed using the *Search History *link. This link can be used to access prior searches or limit search results. For example, one can search first for *casp *as "keyword symbol" and then for mRNAs expressed in the brain, searching by *tissue *for the term *brain*. The queries can be accessed using the *search history *button and can be combined using boolean logic (*AND/OR/NOT/XOR*) to get mRNAs that are caspases *and/or/not/xor *expressed in the brain.

Specific examples of GeneSeer use are given in the next section.

## Discussion

We have addressed three problems in genomic research with this project,

1. Access to genomic information through gene names: Biologists have been struggling with this problem for many years, especially in the genomic era where data on sequences has been piling up at a rapid pace.

2. Mapping sequences to gene names: Data from a variety of sources can be in the form of DNA or protein sequences and it is useful to be able to get back to other resources for the gene to which the sequence fragment belongs.

3. Identification of homologs (both orthologs between species and paralogs within a species) across several species for a given gene: It is difficult to locate orthologs and paralogs, given the name of a gene in one species. It would also be useful to get a quick view of an approximate phylogeny of the set of genes returned.

**Figure 2:**
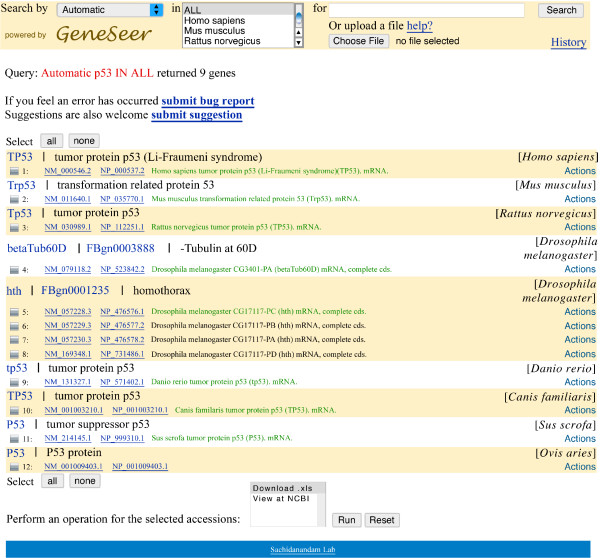
**Results of search for *p53***. GeneSeer results for a search of *p53*. Note the concise and accurate list of genes, which is almost impossible to find on any other website/tool without human curation, based on currently available resources.

**Figure 3:**
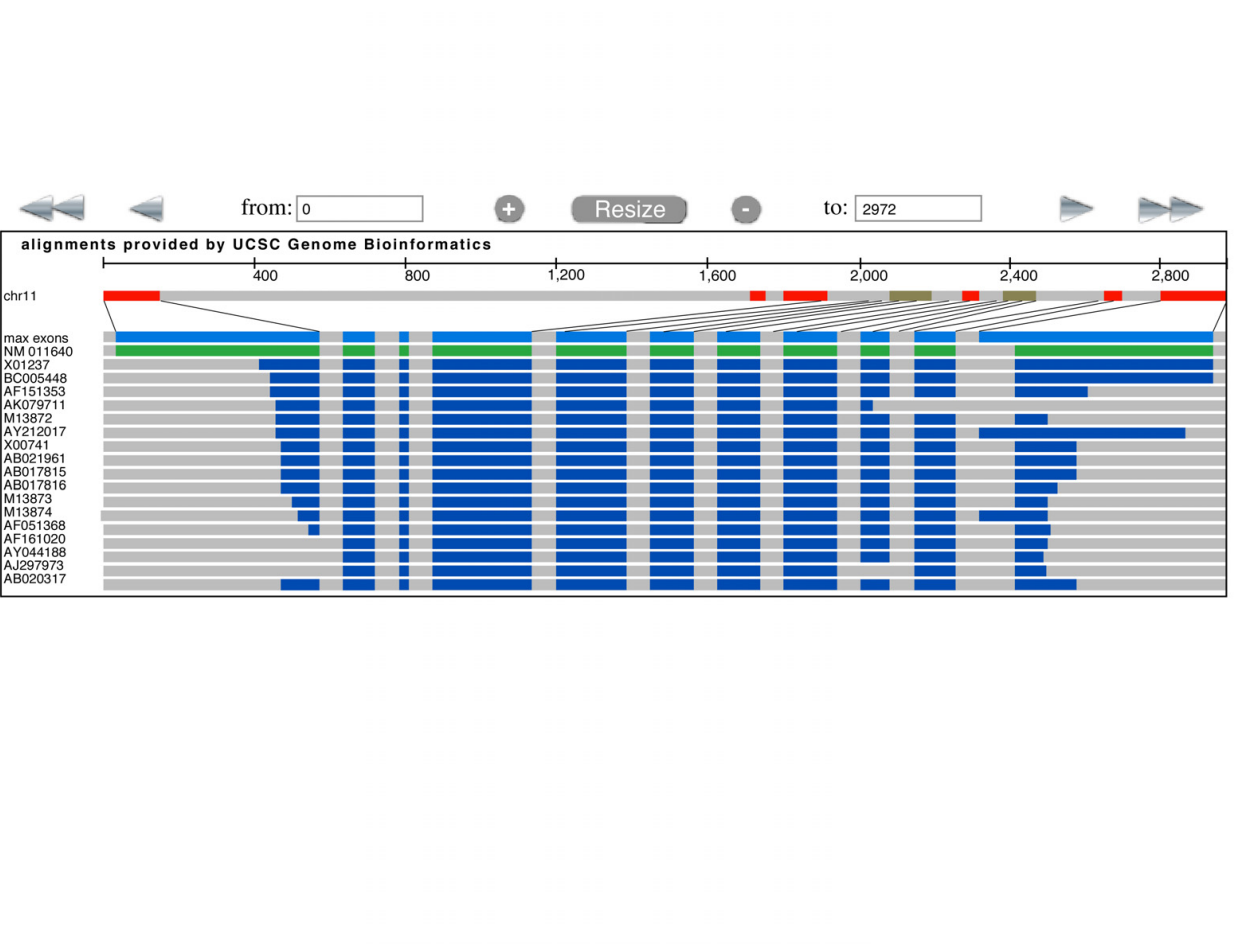
**View of alignment of splice variants of mouse *p53***. A view of the alignments of *p53 *splice variants against the genomic sequence, using *lwgv*, a C-based light weight genome viewer from our lab that is freely available on Source Forge [11].

**Figure 4:**
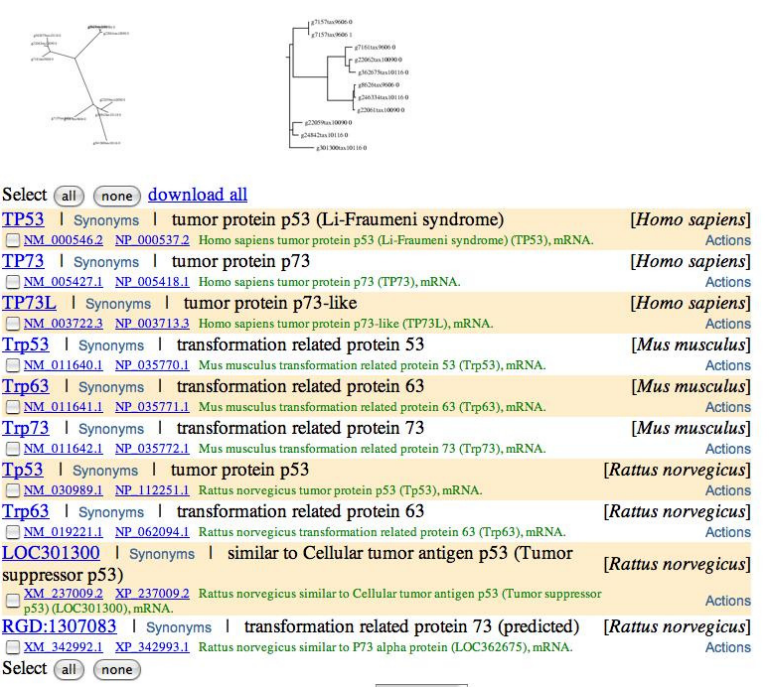
**Homologs of *p53 *and a rudimentary phylogenetic tree**. All the homologs of the *p53 *protein and a thumbnail of a phylogenetic tree constructed from their multiple alignment are shown. The thumbnail links to a bigger picture with more details, as explained in the next figure. The tree gives a rough idea of the phylogenetic relationships between the various proteins and identifies the proteins that need to be analysed further for understanding the evolution of this family.

**Figure 5:**
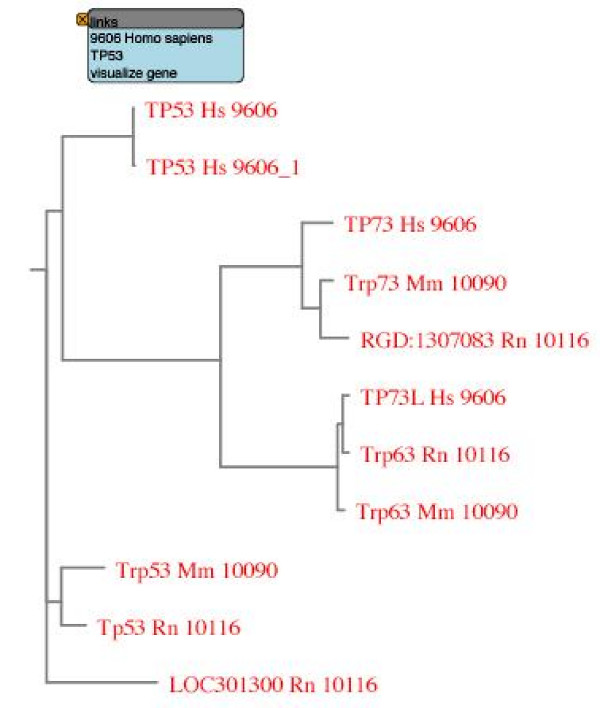
**Detailed view of *p53 *phylogeny**. A more detailed view of the phylogenetic trees seen as thumbnails in the previous figure. Each protein name is followed by the Taxonomy ID for the organism, as specified by NCBI [22]. For example, 9606 is the Taxonomy ID for Homo sapiens. Here, the tree is rendered in SVG format (scalable vector graphics) where each protein name is linked to resources for that gene which appear in a pop-up window on a mouse-over. The pop-up window (the blue box in the figure) can be locked in place by a click on the left-mouse-button. A static jpeg format image is also offered on the website. The SVG image allows control of image magnification through the mouse button.

We use the gene *p53 *to showcase some of the abilities of GeneSeer. Figure 2 shows the result of searching for *p53 *on GeneSeer. Figure 3 shows the alignment of the splice variants of *p53 *accessed through the *Action *menu on the page shown in figure 2. Figures 4 and 5 show a phylogenetic tree for *p53 *generated using the *Action *menu on the page shown in figure 2.

GeneSeer can handle all the examples (ABC1, cyclin D1, hPRL, p53, and PUF60) cited in the introduction. It has information on several model organisms: *H. sapiens*, *M. musculus*, *R. norvegicus*, *D. melanogaster*, *C. elegans*, *S. cerevisiae*, *S. pombe *and *A. thaliana*. It can help visualize splice variants, SNPs and short hairpin RNA silencing constructs (shRNAs, using names from RNAi codex [35], it returns the mRNA that contains the construct) that align with any mRNA. It allows searching for homologs for any gene across species, based on our pairwise sequence alignments of the proteins.

Another example that affords some valuable lessons is the Major Histocompatibility Complex (MHC) region in the human genome [36]. There are several genes in this region that exhibit duplications and also exhibit variations across populations. One such gene, HLA-A (accession [GenBank:NM_002116] can be found through GeneSeer. There are variants such as Aw-80 (accession [GenBank:Q09160], which is in the same locus, but will not appear as a separate gene. In fact, a search on GeneSeer will return HLA-A. To study the variants and the intricacies of this gene family (as well as the immunoglobulin family cited earlier), the best place to start is use a specialized information system such as IMGT [17]. HUGO [5] now assigns names starting with the letters *HCG *to genes in the MHC, one way to search for them in GeneSeer is to use *HCG*% and do an automatic search, but this will return several unrelated hits, a better approach, since we know *HCG *is going to be a part of a gene symbol, is to search by *keyword symbol *with this term.

GeneSeer has some limitations, that necessitate ocassional human intervention. GeneSeer is designed to err on the side of caution. It will find unique answers where possible, but will leave in all ambiguities when a unique resolution is impossible without more information. For example, the symbol nos is used for both *nitric oxide synthase *and *Nanos *in *D. melanogaster*, GeneSeer will return both results. The program will reduce the number of possibilities, so that a human user is not overwhelmed by information. Sometimes human judgement is required to find homologs of genes: For example, while GeneSeer will list all homologs of *staufen*, the human user has to decide which one is interesting from a biological standpoint and if it is a functional homologue, that is, contains the active domains of interest.

### Comparison with other tools

Most of the tools we have been able to find are geared towards controlled vocabularies and aim at reducing the diversity of names. Our viewpoint is different, we start with unique sequences represented in our SOFAR databases for each species, and resolve all names to sequences in this database. We describe some of these papers and tools to give an idea of why GeneSeer is unique.

Some work has been done on identification and disambiguation of gene symbols, we consider one such report here [37] which is a thesaurus-based approach. The method underlying their approach of building a translation table using names from a variety of sources is similar, but their goal is to recognize names in documents and abstracts in order to mine texts, while GeneSeer aims to use gene symbols/names to locate genomic resources and homologs in other species. We also believe our synonym table is much more extensive, since we have identified the gaps in various synonym tables that are available, through intensive field testing.

GeneCards [38] is a database of human genes, their products and their involvement in diseases. It is designed to return concise information on the function of genes and is human-gene centric. A search for p53 results in 925 hits, which are organized into microcards (single line descriptors only) and minicards that have detailed information and are organized by relevance to the search term. The top microcard is the actual p53 gene, and the second one in this list is Mdm2, which interacts with p53. To find Mdm2 through a p53 search in GeneSeer, one would have to search by *Definitions *for the term p53 and it will return p53 and others that interact with p53. Searching for *PUF *as a keyword symbol in GeneSeer returns PUF60/RoBPI/siah-bp while GeneCards returns unrelated genes. The point of this discussion is not to find cases where GeneSeer excels, but to highlight the differences in the abilities of these programs.

Global Gene Hunter [39] is a tool that is a part of the Saccharomyces Genome Database (SGD) [40]. Given a gene name, the site runs searches on six databases, Saccharomyces Genome Database (SGD), PubMed, Entrez Gene, Protein Data Bank Homologs, UniProt [41] and MIPS [42]. The search can be limited to a subset of these databases. The results from each of these databases is returned as part of a large page, but are not organized. It suffers from the failings of the search interfaces provided by these databases and puts the onus of organizing the results on the user.

BioMinT [43], the Gene and Protein Name Synonyms Database, allows the user to find synonyms. A search for PUF60 on this site listed fourteen *H. sapiens *and twenty-one *D. melanogaster *genes and proteins, but they were essentially products of a single gene from each genome. The returned results could have been compressed further.

GeneDB [44] is a resource that provides a portal for access to data generated by pathogen sequencing at several collaborating research centers. Searching for PUF60 found 7 hits in *G. morsitans*, probably from the same gene, the interface is a bit inconvenient and the results were not comprehensive.

DBGET [45] holds information from a variety of databases. But a search for PUF60 failed and a search for p53 returned a long list that was not easily comprehensible.

Thus, GeneSeer is more comprehensive and has an easier interface when compared with other public tools. Its focus and goals are also a bit different from most tools that are currently available online.

## Conclusion

GeneSeer is a powerful engine that is available freely over the web and can be accessed either by a web-browser or by standard programming languages. GeneSeer is an evolving project, there are many more features that can be added, such as sequence and genomic data from new organisms. We plan to add data from F. rubripes [46] in the near future. Prokaryotic gene names are in the system, but it requires additional work to develop SOFAR databases and the homology viewer. User-feedback will be used to prioritize improvements and addition of new features. We want to make GeneSeer compatible with the goals of the semantic web [29] by adding features such as RDF/xml downloads, that will allow it to be indexed and be machine readable. GeneSeer was designed with flexible use in mind, allowing it be freely incorporated into other tools. It is used in several tools developed at Cold Spring Harbor Laboratory (such as the RNAi Codex, an shRNA portal [35]).

## Availability and requirements

The GeneSeer server [28] can be freely accessed over http allowing easy access from any computer with an internet connection and a web-browser. GeneSeer can also be accessed via a programming interface that has been described above.

## Authors' contributions

Ravi Sachidanandam conceived the idea for the project, developed several tools and resources and wrote the manuscript. Andrew Olson is the primary implementer of the ideas, developed the website and also contributed ideas to the design of the tool. Tim Tully suggested the homology tool and identified the biological relevance of the tool.

**Figure 1:**
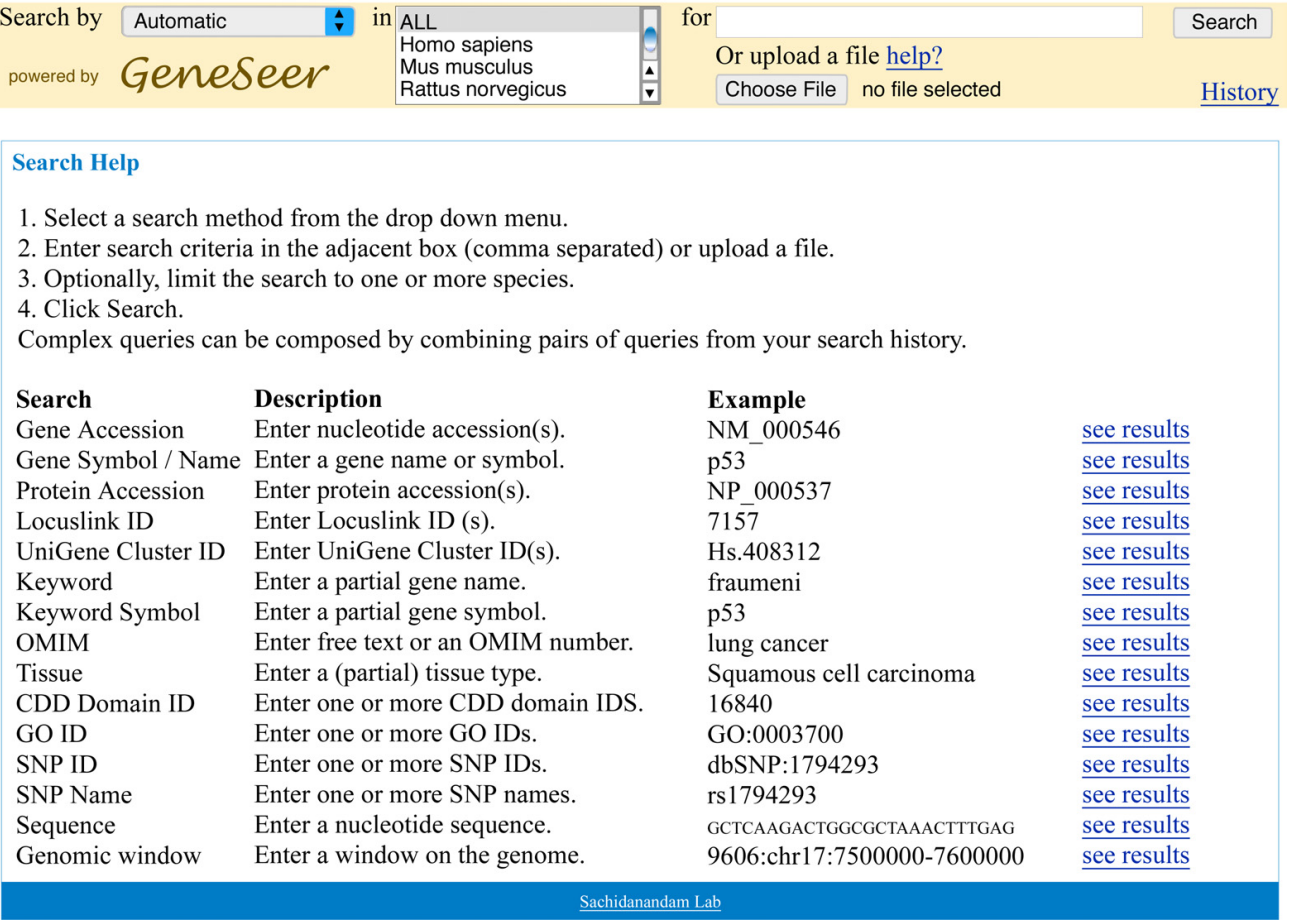
**Web interface for GeneSeer**. The front page of the GeneSeer website.

**Table 1:** Results of *TYRO *keyword symbol search for several species. All the results returned for keyword symbol search for *TYRO *also known as *Eph *are shown here. Some proteins that interact with *TYRO *are also reported.

Keyword Symbol Search *TYRO*				
Number	Species	Accession	GeneID	Definition

1	9606	[GenBank:NM_005233]	2042	Homo sapiens EphA3 (EPH A3), transcript variant 1, mRNA.
2	9606	[GenBank:NM_182644]	2042	Homo sapiens EPH receptor A3 (EPHA3), transcript variant 2, mRNA.
3	9606	[GenBank:NM_004438]	2043	Homo sapiens EphA4 (EPHA4), mRNA.
4	9606	[GenBank:NM_004439]	2044	Homo sapiens EphA5 (EPHA5), transcript variant 1, mRNA.
5	9606	[GenBank:NM_182472]	2044	Homo sapiens EphA5 (EPHA5), transcript variant 2, mRNA.
6	9606	[GenBank:NM_004442]	2048	Homo sapiens EphB2 (EPHB2), transcript variant 2, mRNA.
7	9606	[GenBank:NM_017449]	2048	Homo sapiens EphB2 (EPHB2), transcript variant 1, mRNA.
8	9606	[GenBank:NM_004443]	2049	Homo sapiens EphB3 (EPHB3), mRNA.
9	9606	[GenBank:NM_004444]	2050	Homo sapiens EphB4 (EPHB4), mRNA.
10	9606	[GenBank:NM_006182]	4921	Homo sapiens discoidin domain receptor family, member 2 (DDR2), mRNA.
11	9606	[GenBank:NM_000372]	7299	Homo sapiens tyrosinase (oculocutaneous albinism IA) (TYR), mRNA.
12	9606	[GenBank:NM_006293]	7301	Homo sapiens TYRO3 protein tyrosine kinase (TYRO3), mRNA.
13	9606	[GenBank:X72887]	7302	H. sapiens TYRO3P mRNA.
14	9606	[GenBank:NM_003332]	7305	Homo sapiens TYRO protein tyrosine kinase binding protein (TYROBP), transcript variant 1, mRNA.
15	9606	[GenBank:NM_198125]	7305	Homo sapiens TYRO protein tyrosine kinase binding protein (TYROBP), transcript variant 2, mRNA.
16	10090	[GenBank:NM_010140]	13837	Mus musculus Eph receptor A3 (Epha3), mRNA.
17	10090	[GenBank:NM_007936]	13838	Mus musculus Eph receptor A4 (Epha4), mRNA.
18	10090	[GenBank:NM_010142]	13844	Mus musculus Eph receptor B2 (Ephb2), mRNA.
19	10090	[GenBank:NM_010143]	13845	Mus musculus Eph receptor B3 (Ephb3), mRNA.
20	10090	[GenBank:NM_010144]	13846	Mus musculus Eph receptor B4 (Ephb4), mRNA.
21	10090	[GenBank:NM_022563]	18214	Mus musculus discoidin domain receptor family, member 2 (Ddr2), mRNA.
22	10090	[GenBank:NM_019392]	22174	Mus musculus TYRO3 protein tyrosine kinase 3 (Tyro3), mRNA.
23	10090	[GenBank:NM_011662]	22177	Mus musculus TYRO protein tyrosine kinase binding protein (Tyrobp), mRNA.
24	10090	[GenBank:NM_009465]	26362	Mus musculus AXL receptor tyrosine kinase (Axl), mRNA.
